# Liquid-Phase Synthesis of Lithium Argyrodite Sulfide Electrolytes Using Tetrahydrofuran and Water

**DOI:** 10.1021/acs.langmuir.5c06765

**Published:** 2026-05-30

**Authors:** Takashi Hashii, Hayata Tanigaki, Tomohiro Furukawa, Hiroe Kowada, Kota Motohashi, Atsushi Sakuda, Akitoshi Hayashi

**Affiliations:** † Department of Applied Chemistry, Graduate School of Engineering, 12936 Osaka Metropolitan University, 1-1 Gakuen-cho, Naka-ku, Sakai, Osaka 599-8531, Japan; ‡ Institute for Materials Research, Tohoku University, 2-1-1 Katahira, Aoba-ku, Sendai, Miyagi 980-8577, Japan

## Abstract

Li_6_PS_5_Cl argyrodite sulfide electrolytes exhibit high ionic conductivity, making them promising candidate electrolyte materials for all-solid-state batteries. Although water is an environmentally friendly and attractive solvent, its use in the synthesis of Li_6_PS_5_Cl has been limited due to the low moisture stability of Li_6_PS_5_Cl. In this study, Li_6_PS_5_Cl argyrodite sulfide electrolytes were synthesized via liquid-phase synthesis using water as the main solvent through optimization of both the synthesis conditions and the composition of the Li_3_PS_4_·Li_2_S·LiCl precursor. In this process, the precursor is formed via the aqueous phase, followed by heat treatment that drives the crystallization of Li_6_PS_5_Cl and improves its crystallinity. A series of electrolyte samples derived from precursors with varying Li_3_PS_4_ contents, *x*Li_3_PS_4_·Li_2_S·LiCl (*x* = 1.0–1.4), were systematically prepared and characterized, revealing their structural and electrochemical properties. The electrolyte derived from the optimal precursor composition (*x* = 1.2) exhibited a high ionic conductivity of 1.2 × 10^–3^ S cm^–1^ at 25 °C in a green compact, and the all-solid-state cells assembled using this electrolyte demonstrated reversible charge–discharge behavior over 100 cycles at room temperature. These results demonstrate that water can be successfully used as the main solvent to synthesize argyrodite-type sulfide electrolytes and provide a versatile strategy for sustainable production of high-performance sulfide solid electrolytes suitable for practical use.

## Graphical abstract

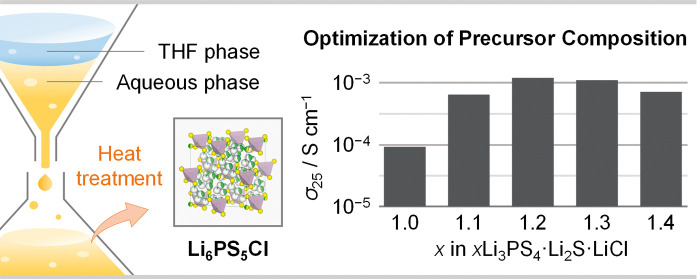

## Introduction

Sulfide solid electrolytes have emerged as promising candidate electrolyte materials for high-performance all-solid-state batteries due to their high ionic conductivity and favorable mechanical properties that enable densification through simple pressing.^1,2^ However, the sensitivity of sulfide electrolytes to hydrolysis and oxidation upon exposure to water has traditionally limited the use of aqueous media in their synthesis. Consequently, to obtain sulfide solid electrolytes, conventional methods have mainly relied on high-temperature solid-state reactions, energy-intensive mechanochemical approaches, or liquid-phase syntheses using organic solvents.^3–5^

Liquid-phase synthesis using organic solvents has attracted attention as a relatively rapid method with potential for scalable production. In the synthesis process, the raw materials are dissolved or dispersed in an organic solvent, followed by stirring and the corresponding chemical reaction. The solvent is subsequently removed through drying and heat treatment to obtain the desired product.^5^ However, the environmental impact of organic solvents poses a significant challenge for the wide use of this method, motivating the development of alternative strategies that combine scalability and sustainability. In particular, synthesis routes that use water as the main solvent offer an environmentally friendly and scalable pathway for the fabrication of sulfide solid electrolytes. Our previous studies have demonstrated that representative sulfide electrolytes, such as Li_10_MP_2_S_12_ (M = Ge, Sn), can be successfully synthesized via liquid-phase synthesis using water as the main solvent.^6–8^ This finding indicates that the synthesis of sulfide electrolytes in water, previously regarded as infeasible, can in fact be realized under appropriately designed conditions, thereby opening new avenues for the scalable production of sulfide solid electrolytes.

Among sulfide solid electrolytes, argyrodite-type Li_6_PS_5_X (X = Cl, Br, I) have attracted considerable attention due to their high ionic conductivities (typically on the order of 10^–3^–10^–2^ S cm^–1^) and moderate electrochemical stability,^9–13^ making these materials promising candidates for use in all-solid-state battery applications. Previous studies on the synthesis of Li_6_PS_5_X electrolytes have primarily focused on solid-state reactions^14,15^ or liquid-phase syntheses using organic solvents such as alcohols,^16,17^ ethers,^18^ nitriles,^19^ amines,^20^ or mixed solvents,^21–23^ whereas the use of water as the main solvent for the preparation of these electrolytes has not been explored due to their sensitivity to water. In particular, Li_6_PS_5_Cl electrolytes are known to readily decompose upon exposure to moisture, forming secondary phases such as LiCl, LiOH, Li_3_PO_4_, Li_2_SO_4_, Li_2_S, and Li_2_CO_3_,^24–26^ highlighting their relatively poor moisture stability and making the synthesis of these materials using water as a solvent particularly challenging.

Directly addressing this problem, in this study, Li_6_PS_5_Cl electrolytes were synthesized via liquid-phase synthesis using water as the main solvent. In this process, the precursor is formed via the aqueous phase, followed by heat treatment that drives the crystallization of Li_6_PS_5_Cl and improves its crystallinity. Although complete stability in water cannot be assumed, the PS_4_^3–^ tetrahedral units exhibit relatively high hydrolysis tolerance compared with other thiophosphate species.^27^ Therefore, preformed PS_4_^3–^ tetrahedral species were introduced into the aqueous phase. In addition, to compensate for possible phosphorus oxidation during the process, a series of samples with nominal compositions *x*Li_3_PS_4_·Li_2_S·LiCl (*x* = 1.0–1.4) were systematically prepared to investigate the effect of Li_3_PS_4_ content on their structure and electrochemical properties, and successful synthesis of Li_6_PS_5_Cl solid electrolytes with favorable properties was achieved. This approach demonstrates the potential of using water in the synthesis of argyrodite-type sulfide electrolytes and provides a versatile strategy for the scalable preparation of sulfide solid electrolytes.

## Experimental Section

### Synthesis

The synthesis of Li_6_PS_5_Cl electrolytes was adapted from our previously reported liquid-phase synthesis of Li_10_GeP_2_S_12_ using tetrahydrofuran (THF) and water as solvents.^6^ The overall synthetic scheme developed in the current study is illustrated in Figure 1. Li_6_PS_5_Cl electrolytes were prepared by combining a Li_2_S–LiCl aqueous solution with a Li_3_PS_4_–THF suspension prepared according to the previously reported procedure.^28^ To prepare the Li_2_S–LiCl aqueous solution, the starting materials, Li_2_S (99.9%, Mitsuwa Chemical) and LiCl (99.0%, FUJIFILM Wako Pure Chemical) were weighed and dissolved in ion-exchanged water and then stirred at room temperature for 24 h under atmospheric conditions. Separately, Li_2_S and P_2_S_5_ (99%, Sigma-Aldrich) were weighed in the desired amounts, THF (99.5%, FUJIFILM Wako Pure Chemical) was added, and the mixture was stirred at room temperature for 24 h under dry Ar atmosphere to prepare the Li_3_PS_4_–THF suspension. Li_2_S, P_2_S_5_, and LiCl were weighed in stoichiometric amounts to yield 2.0 g of the solid electrolyte. P_2_S_5_ and an amount of Li_2_S corresponding to 3 molar equivalents relative to P_2_S_5_ were added to 20 g of THF, while the remaining Li_2_S and LiCl were dissolved in 20 g of water. Then, the obtained suspension and aqueous solution were rapidly mixed in a separatory funnel for several tens of seconds under atmospheric conditions to minimize side reactions such as hydrolysis-induced oxidation, followed by several minutes of extraction before separation of the aqueous phase. The aqueous phase was then dried under vacuum at 200 °C for 3 h and then heated at 550 °C for 2 h under dry Ar atmosphere to obtain the powdered sample. During the heat treatment processes, the temperature was increased at a rate of 10 °C min^–1^ until reaching 500 °C, and then was increased further at a rate of 1 °C min^–1^ until reaching 550 °C. This procedure was used to prepare samples with the nominal composition *x*Li_3_PS_4_·Li_2_S·LiCl (*x* = 1.0–1.4). In these samples, *x* = 1.0 corresponds to the reference composition of Li_6_PS_5_Cl, and increasing *x* signifies increased Li_3_PS_4_ content. This procedure was also applied to prepare samples with the nominal composition 1.2Li_3_PS_4_·Li_2_S·LiX (X = Br, I) to examine the wider applicability of the developed process to Li_6_PS_5_X electrolytes with halogens other than chlorine. In these samples, either LiBr (99.9%, Kojundo Chemical) or LiI (99.999%, Sigma-Aldrich) were used instead of LiCl, while all other steps were the same as those for the Cl-containing sample.

**Figure 1:**
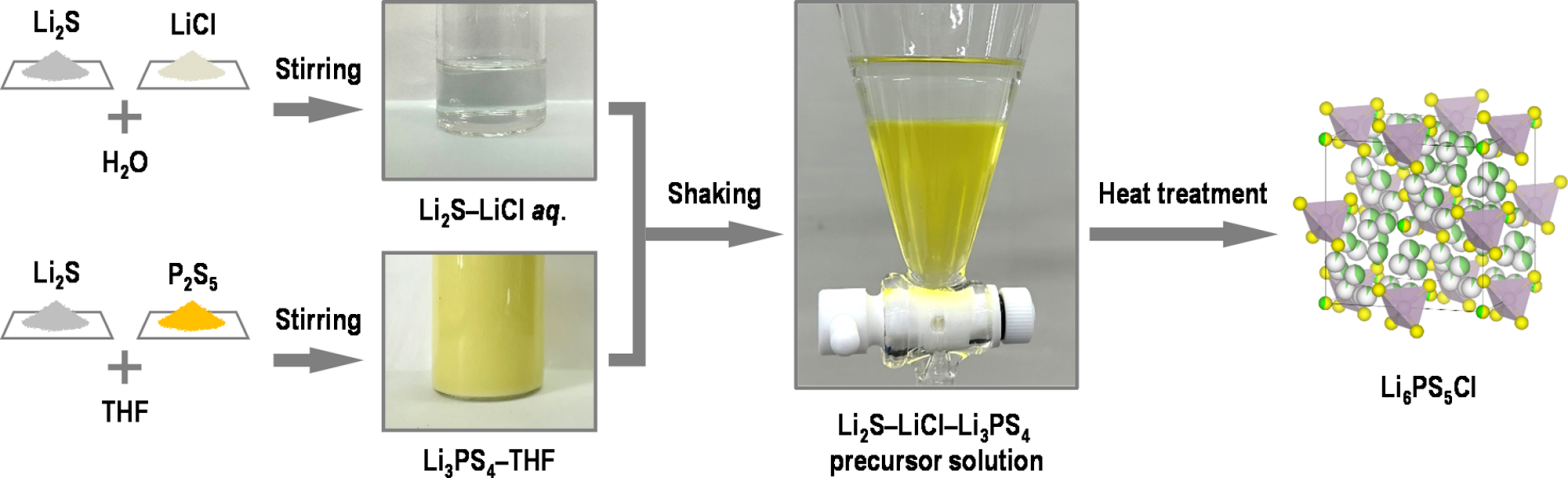
Schematic illustration of the synthetic route for Li_6_PS_5_Cl electrolytes using THF and water as solvents. The crystal structure of Li_6_PS_5_Cl was drawn using VESTA software.^29^

### Material Characterization

To examine the sample structures, X-ray diffraction (XRD) measurements were performed (Rigaku SmartLab XRD; Rigaku) using CuKα radiation in the 2θ range of 10–60° at a scan rate of 10° min^–1^. Additionally, Raman spectroscopy measurements were carried out (RAMANtouch spectrometer; Nanophoton) using a 532 nm laser. Thermogravimetry mass spectrometry (TG-MS) was conducted using a gas chromatography-mass spectrometer (JMS-Q1500GC; JEOL) coupled with thermogravimetric and differential thermal analyses (STA 2500 Regulus; Netzsch). The furnace was heated from 25 to 500 °C at a rate of 10 °C min^–1^ with the sample placed in an Al pan under flowing dry He gas. The evolved gases were introduced into the ion source of the mass spectrometer, which was operated at an electron energy of 70 eV and a mass range of 10–200, with the ion source temperature set to 200 °C. Elemental analysis (CHNS) was performed using an elemental analyzer (Vario EL cube; Elementar Analysensysteme). ^31^P and ^1^H nuclear magnetic resonance (NMR) spectroscopy were performed (JNM-ECZL 500G spectrometer; JEOL) on the THF phase obtained during synthesis, which was filled into glass capillaries under atmospheric conditions. ^31^P NMR spectra were recorded at a frequency of 202.5 MHz using a π/2 pulse length of 3.25 μs and a recycle delay of 600 s, while rotating at 15 Hz, with chemical shifts referenced to aqueous H_3_PO_4_. ^1^H NMR spectra were recorded at 500 MHz using a π/2 pulse length of 7.1 μs and a recycle delay of 600 s, while rotating at 15 Hz, with chemical shifts referenced to tetramethylsilane (TMS). ^31^P magic-angle-spinning nuclear magnetic resonance (^31^P MAS NMR) spectroscopy was conducted on powdered samples, which were packed into zirconia spinners under dry Ar atmosphere using the same instrument. The spectra were recorded at 202.5 MHz using a π/2 pulse length of 3.25 μs and a recycle delay of 600 s, while rotating at 20 kHz, and chemical shifts were externally referenced to aqueous H_3_PO_4_. Additionally, scanning electron microscopy (SEM) observations were carried out using a field-emission scanning electron microscope (FE-SEM, SU8220; Hitachi High-Technologies) with an energy-dispersive X-ray spectrometer (EDX, EMAXEvolution X-MAX; Horiba). The relative density was determined using a gas pycnometer (AccuPyc II 1340; Shimadzu) under dry Ar atmosphere by calculating the ratio of the pellet density to the powder density of the sample. For comparison, commercially available Li_6_PS_5_Cl (Sigma-Aldrich) was used as a reference sample prepared by conventional methods.

### Electrochemical Characterization

The ionic conductivities of the prepared samples were measured using an impedance analyzer (SI-1260; Solartron Analytical). The sample powders were pelletized using a uniaxial press at 360 MPa for 5 min at room temperature. Both sides of the pellets were then sputtered with thin gold films to produce ion-blocking electrodes. The pellets were sealed in a laminate-type pouch cell to prevent air exposure. The activation energy (*E*_a_) of ionic conduction was calculated using the Arrhenius equation: σ = *A* exp­(−*E*_a_/*RT*), where σ, *A*, *R*, and *T* are the ionic conductivity, pre-exponential factor, gas constant, and absolute temperature, respectively.

The composite electrodes for linear sweep voltammetry (LSV) measurements were prepared by hand mixing the solid electrolyte synthesized from the 1.2Li_3_PS_4_·Li_2_S·LiCl precursor and vapor-grown carbon fiber (VGCF, Resonac) in a weight ratio of 70:30. For the charge–discharge measurements, the composite positive electrodes were prepared by hand mixing 1 wt % LiNbO_3_-coated LiNi_1/3_Co_1/3_Mn_1/3_O_2_ (NCM)^30,31^ and the same solid electrolyte in a weight ratio of 70:30. The cells were fabricated as follows. The synthesized 1.2Li_3_PS_4_·Li_2_S·LiCl solid electrolyte (80 mg), used as the solid electrolyte layer, was placed in a polycarbonate tube (diameter: 10 mm) and pressed at 36 MPa. The prepared composite electrode was then placed on the solid electrolyte layer and pressed at 360 MPa for 5 min, and a Li foil (thickness: 0.25 mm, diameter: 7 mm) and an In foil (thickness: 0.3 mm, diameter: 8 mm) were placed on the other side of the solid electrolyte layer. Then, stainless-steel disks were installed as current collectors on each side of the prepared pellet, and the cells were tightly stacked. All processes were performed under dry Ar atmosphere. LSV measurements were conducted using an electrochemical workstation (Celltest 1470E; Solartron Analytical) at 25 °C. Charge–discharge measurements were conducted using a charge–discharge measuring device (BTS-2004; Nagao) at 25 °C within a voltage range of 1.9–3.8 V vs Li–In.

## Results and Discussion

Liquid-phase synthesis of Li_10_GeP_2_S_12_ electrolytes using THF and water as solvents has been reported in our previous work.^6^ In the current study, this synthesis process was adapted for the preparation of Li_6_PS_5_Cl electrolytes by combining a Li_2_S–LiCl aqueous solution with a Li_3_PS_4_–THF suspension. It should be noted that, during the preparation of the Li_2_S–LiCl aqueous solution, Li_2_S rapidly dissolves in water and forms a strongly basic solution, where sulfide species are stabilized. Under such basic conditions, the acid–base equilibrium between H_2_S and HS^–^ is shifted toward HS^–^, and thus continuous H_2_S release during stirring is not expected.^6,7,32^

Figure 2a shows the XRD patterns of the synthesized samples. The bottom pattern corresponds to the sample prepared with the stoichiometric composition (*x* = 1.0). Li_6_PS_5_Cl was observed as the main phase, while Li_3_PO_4_ and the starting materials (Li_2_S and LiCl) were detected as secondary phases. The formation of Li_3_PO_4_ as a secondary phase is attributed to the partial oxidation of Li_3_PS_4_ by water, which can be described by

1 $${Li}_{3}{PS}_{4}+{4H}_{2}O\to {Li}_{3}{PO}_{4}+{4H}_{2}S$$

**Figure 2:**
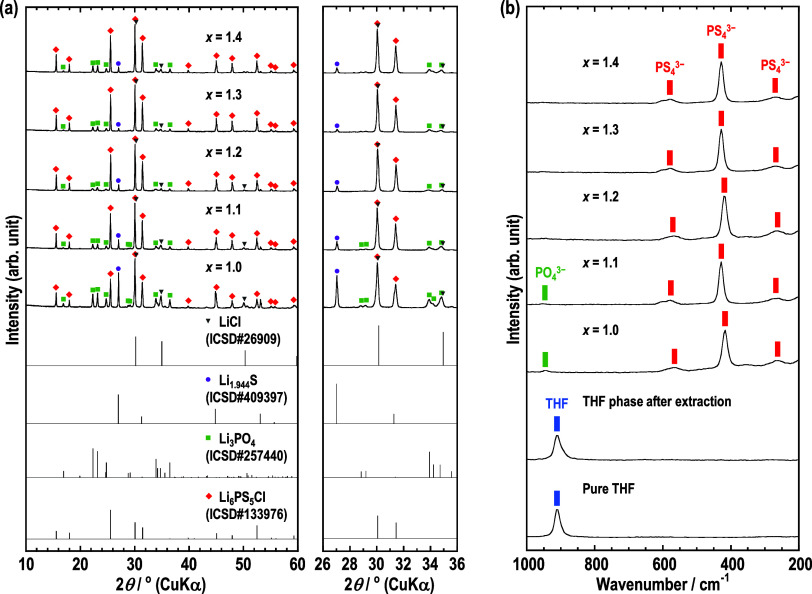
(a) XRD patterns and (b) Raman spectra of the Li_6_PS_5_Cl electrolyte samples synthesized via liquid-phase synthesis from *x*Li_3_PS_4_·Li_2_S·LiCl (*x* = 1.0–1.4) precursors using THF and water as solvents. The Raman spectrum of the extracted THF phase is also presented in (b).

This reaction can be rationalized based on the HSAB principle, where phosphorus, being a relatively hard acid compared with germanium, tin, and antimony, tends to form strong bonds with oxygen.^8,33,34^

Since Li_6_PS_5_Cl is obtained from Li_3_PS_4_, Li_2_S, and LiCl according to the nominal reaction

2 $${Li}_{3}{PS}_{4}+{Li}_{2}S+LiCl\to {Li}_{6}{PS}_{5}Cl$$

the conversion of Li_3_PS_4_ to Li_3_PO_4_ results in a loss of Li, P, and S required to form the target Li_6_PS_5_Cl phase, leaving residual Li_2_S and LiCl. To compensate for this loss, the starting composition was adjusted by increasing the amount of Li_3_PS_4_ (*x*Li_3_PS_4_·Li_2_S·LiCl, *x* = 1.0–1.4). Among various thiophosphate units, PS_4_^3–^ is known to exhibit relatively high stability against moisture.^27^ Based on this, rather than supplementing the Li, P, and S consumed due to the partial conversion of Li_3_PS_4_ to Li_3_PO_4_ with additional P_2_S_5_ or other sulfur sources, the compensation was achieved by increasing the amount of Li_3_PS_4_ itself, taking advantage of the high moisture stability of PS_4_^3–^. As shown in Figure 2a, the intensity of the peaks corresponding to the residual starting materials decreases for samples with *x* ≥ 1.1 compared to that of the *x* = 1.0 sample, indicating effective compensation. All synthesized samples (*x* = 1.0–1.4) exhibit XRD peak positions nearly identical to those of commercial Li_6_PS_5_Cl (Sigma-Aldrich, 99.5%) (Figure S1), with only minor variations, indicating that the main crystal structure is consistent with conventional samples, although small amounts of impurities are present in the synthesized materials. The full width at half-maximum (FWHM) of the main peak near 25.5° is slightly broader (∼0.092–0.10°) than that of the commercial sample (∼0.073°), suggesting a modest decrease in crystallinity.

Figure 2b displays the Raman spectra of all samples (*x* = 1.0–1.4), in which bands assigned to PS_4_^3–^ were clearly observed at ∼ 270, 420, and 550 cm^–1^. The lower spectrum corresponds to the THF phase obtained after extraction, with the spectrum of pure THF also shown for comparison. The absence of the PS_4_^3–^ bands in the extracted THF phase confirms that the Li_3_PS_4_ component was effectively transferred from the THF phase to the aqueous phase during the separation process. Additional ^31^P and ^1^H NMR analyses were conducted for the extracted THF phase (Figures S2 and S3). The ^31^P NMR spectrum shows only weak signals close to the background level, indicating that phosphorus-containing species are scarcely present in the THF phase. The ^1^H NMR spectrum confirms the presence of residual water in the THF phase due to partial mutual solubility during extraction, with a molar ratio of THF/H_2_O = 1.0:0.48. Because the THF phase can be easily recovered, the release of organic solvent into the environment can be minimized, potentially reducing the environmental impact and providing guidance for the design of more sustainable processes. A photograph of the THF phase obtained after the extraction, used for Raman spectroscopy and NMR measurements, is provided for reference in Figure S4.

The present synthesis process involves transfer of thiophosphate species into the aqueous phase through a salt-induced THF/water biphasic system generated during extraction. Salt-induced liquid–liquid phase separation and solute partitioning in aqueous biphasic systems have recently attracted increasing attention in interfacial chemistry because such biphasic solvent environments can provide unique media for selective extraction, ion transfer, and materials processing.^35–38^ In our recent study, aqueous processing concepts were successfully extended to the synthesis of sulfide solid electrolytes, including Li_10_SnP_2_S_12_-related materials, demonstrating new processing strategies for water-sensitive sulfide electrolytes through appropriately designed aqueous environments.^6,7^ Building on these studies, the present work further expands aqueous processing of sulfide electrolytes by utilizing salt-induced THF/water biphasic solvent behavior for the synthesis of argyrodite electrolytes.

Figure 3a shows the temperature dependence of the ionic conductivity for the synthesized samples, as determined by AC impedance measurements, and Figure 3b presents the composition dependence of the ionic conductivity at 25 °C, with the corresponding values summarized in Table S1. It is observed that the *x* = 1.0 sample exhibited an ionic conductivity of 9.2 × 10^–5^ S cm^–1^ at 25 °C and increasing the Li_3_PS_4_ content enhanced the conductivity. In particular, the *x* = 1.2 sample achieved a relatively high conductivity of 1.2 × 10^–3^ S cm^–1^, approximately 1 order of magnitude higher than that of the *x* = 1.0 sample. This improvement in the ionic conductivity can be attributed to the more complete formation of the Li_6_PS_5_Cl phase, as indicated by the reduced intensities of the signals of the residual Li_2_S and LiCl in the XRD patterns. The nearly constant activation energy across various compositions further suggests a correlation between the increase in the ionic conductivity and the amount of impurity phases. These results support the rational design of the starting composition in this study in which an increase in the Li_3_PS_4_ content is used to compensate for losses of Li, P, and S during synthesis and promote the formation of the target Li_6_PS_5_Cl phase.

**Figure 3:**
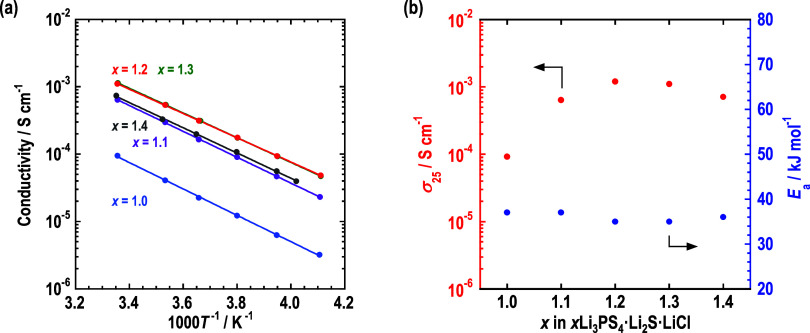
(a) Temperature dependence of the ionic conductivities and (b) composition dependence of ionic conductivities at 25 °C and activation energies of the Li_6_PS_5_Cl samples obtained using *x*Li_3_PS_4_·Li_2_S·LiCl (*x* = 1.0–1.4) precursors.

To further investigate the *x* = 1.2 sample, the thermal behavior of the precursor powder prior to heat treatment at 550 °C was examined by TG–MS (Figure S5). The analysis revealed that H_2_O, H_2_S, CO_2_, and S evolved sequentially upon heating, with water released in two distinct temperature regions. The water released at the lower temperature may originate from the moisture adsorbed on the sample due to its exposure to air prior to the measurement. The evolution of CO_2_ may be related to the decomposition of carbonate species (CO_3_^2–^) formed during synthesis or prior to the measurement, and similar behavior has been reported previously.^25^ The evolution of H_2_S, CO_2_, and S followed the high-temperature water release above ∼ 200 °C, indicating that partial hydrolysis of the precursor may occur in this temperature range and potentially affect the subsequent phase formation.

Figure 4a shows the ^31^P MAS NMR spectrum of the heat-treated *x* = 1.2 sample. The peaks at 85, 36, and 9 ppm were assigned to PS_4_^3–^ in Li_6_PS_5_Cl, and to PO_3_S and PO_4_^3–^ in the oxide impurities, respectively.^9,39^ Peak integration indicates that ∼ 30% of the total phosphorus was oxidized to PO_4_^3–^. It should be noted that this value does not directly correspond to the molar fraction of Li_3_PO_4_ in the overall composition. SEM-EDX analysis of this sample (Figure 4b) revealed particles on the order of several micrometers in size and partial segregation of oxygen. This observation is consistent with the formation of Li_3_PO_4_ as confirmed by the XRD patterns and ^31^P MAS NMR measurements. SEM-EDX images of the heat-treated samples with other compositions are presented in Figure S6, while structural changes of the *x* = 1.2 sample before and after the heat treatment are shown in Figure S7. XRD patterns of the precursor and heat-treated samples (Figure S7a) indicate that while the precursor powder contained LiCl and several unidentified phases, Li_6_PS_5_Cl was formed as the main phase after heat treatment at 550 °C, with a small amount of Li_3_PO_4_. This finding is further supported by the ^31^P MAS NMR spectra of the precursor and heat-treated samples (Figure S7b) as the peak at 9 ppm corresponding to PO_4_^3–^ was absent prior to heat treatment and then only appeared after the heat treatment. Furthermore, the temperature dependence of ionic conductivity for the *x* = 1.2 sample before and after heat treatment is shown in Figure S8. The ionic conductivity was 2.8 × 10^–6^ S cm^–1^ prior to heat treatment and increased substantially to 1.2 × 10^–3^ S cm^–1^ after heat treatment, confirming a significant enhancement in ionic transport upon crystallization of Li_6_PS_5_Cl.

**Figure 4:**
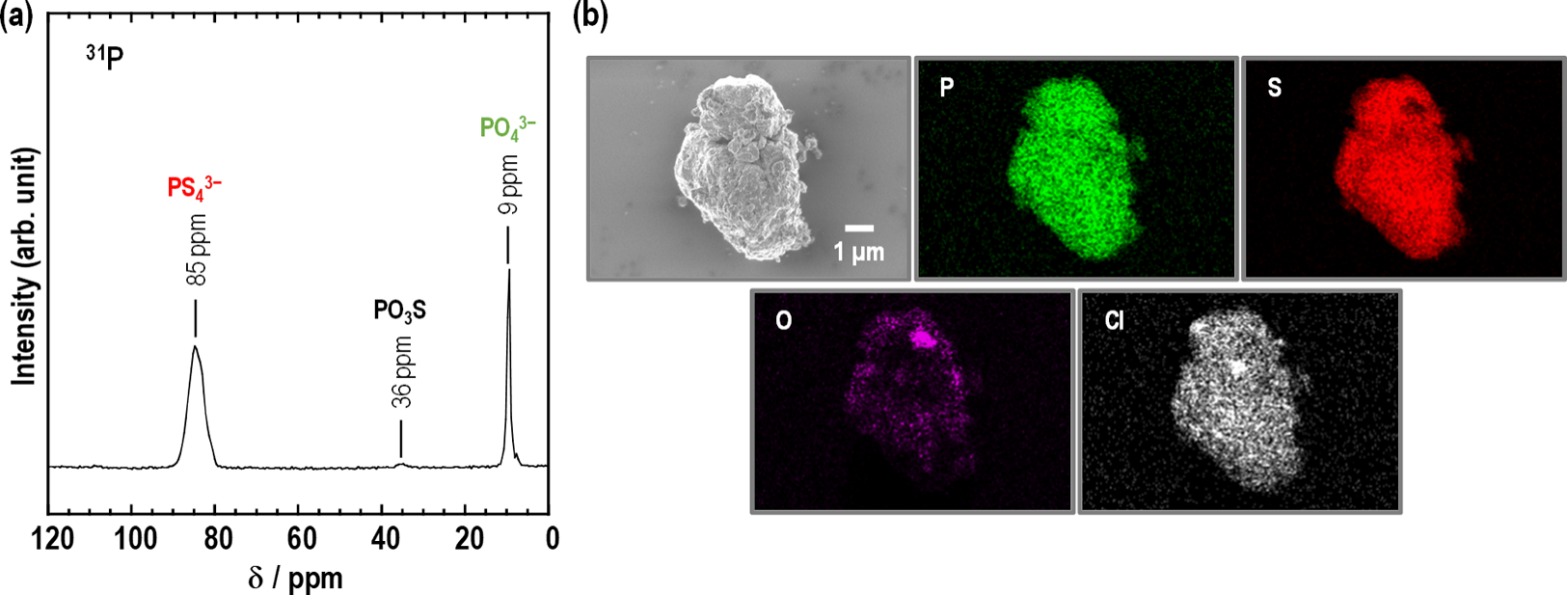
(a) ^31^P MAS NMR spectrum and (b) SEM image and EDX elemental mapping of the Li_6_PS_5_Cl samples obtained using the 1.2Li_3_PS_4_·Li_2_S·LiCl precursor.

Table S2 shows CHNS elemental analysis of the *x* = 1.2 sample before and after heat treatment. For comparison, results for the starting materials simply mixed in a mortar are also presented. The sulfur content of the precursor powder (∼47 wt %) was already lower than that of the simple mortar-mixed starting materials (∼59 wt %), suggesting that oxygen had been incorporated prior to heat treatment. After heat treatment, the sulfur content changed relatively slightly to ∼ 44 wt %, suggesting oxygen incorporation is likely to have occurred mainly during the mixing or drying process. In addition, the hydrogen content decreased from ∼ 1 wt % in the precursor to ∼ 0.6 wt % after heat treatment (with ∼ 0.5 wt % hydrogen detected in the starting materials, possibly near the detection limit), indicating that most protonic species were removed during drying and high-temperature treatment. Collectively, although the chemical state of oxygen cannot be conclusively identified at this stage, the H_2_O evolution observed in TG-MS and the CHNS analysis suggest the presence of oxygen- and hydrogen-containing species in the system. Upon heating, these species may induce hydrolysis of the precursor, contributing to the formation of oxide impurities.

Although Li_3_PO_4_ generally exhibits low ionic conductivity, the samples synthesized in this study still show relatively high ionic conductivities exceeding 10^–3^ S cm^–1^, suggesting that the presence of this secondary phase is unlikely to significantly limit the overall ion transport, while the formation of PO_4_^3–^ species should contribute to the decrease in ionic conductivity at least to some extent. In fact, the ionic conductivity of the heat-treated *x* = 1.2 sample (1.2 × 10^–3^ S cm^–1^) is comparable to previously reported argyrodite samples prepared by liquid-phase synthesis using organic solvents (typically 1–3 × 10^–3^ S cm^–1^)^18–20,22,23^ and to commercially available Li_6_PS_5_Cl (Sigma-Aldrich), which exhibited 1.6 × 10^–3^ S cm^–1^ under the same measurement conditions as in this study (Figure S9). To evaluate the electrochemical stability, LSV of the cell (Li–In/1.2Li_3_PS_4_·Li_2_S·LiCl/1.2Li_3_PS_4_·Li_2_S·LiCl–carbon), where carbon was added to enhance the redox response, was conducted at a scan rate of 1 mV s^–1^ at 25 °C, showing an oxidation current from ∼ 1.8 V vs Li–In and a reduction current from ∼ 1.3 V vs Li–In (Figure S10).

To explore the versatility of the developed synthesis process, 1.2Li_3_PS_4_·Li_2_S·LiX (X = Br, I) samples were also prepared. XRD patterns of these samples after heat treatment (Figure S11) confirmed that Li_6_PS_5_X (X = Br, I) was formed as the main phase with only minor impurities, demonstrating that the developed synthesis process can be successfully extended to argyrodite-type electrolytes containing other halogen elements. The temperature dependence of the ionic conductivity for these samples is presented in Figure S12, with the corresponding values listed in Table S3.

Finally, an all-solid-state cell was fabricated using the *x* = 1.2 sample as the solid electrolyte. Figure 5a displays the charge–discharge curves, and Figure 5b presents the corresponding cycle performance of the cell (Li–In/1.2Li_3_PS_4_·Li_2_S·LiCl/NCM–1.2Li_3_PS_4_·Li_2_S·LiCl) measured at a current density of 0.13 mA cm^–2^ at 25 °C. The cell exhibits stable and reversible electrochemical behavior over 100 cycles. Additionally, its rate performance was evaluated at 25 °C, and moderate capacity was obtained at higher current densities (Figure S13). These results indicate that the *x* = 1.2 sample can function as a solid electrolyte in an all-solid-state cell and has potential for practical applications.

**Figure 5:**
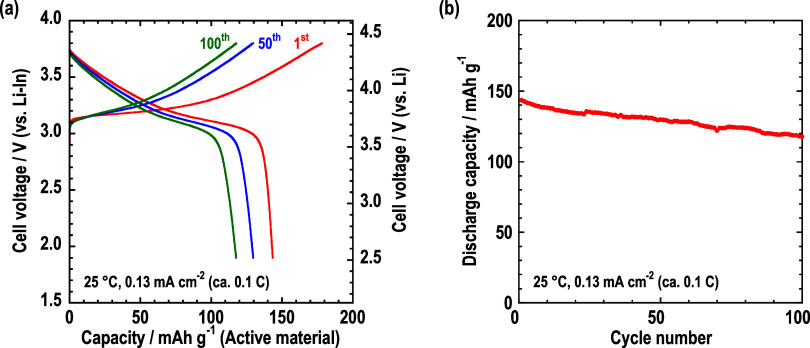
(a) Charge–discharge curves and (b) cycle performance of the all-solid-state cell using the solid electrolyte obtained with the 1.2Li_3_PS_4_·Li_2_S·LiCl precursor.

## Conclusion

In this study, Li_6_PS_5_Cl solid electrolytes were successfully synthesized via liquid-phase synthesis using THF and water as solvents. Li_6_PS_5_Cl was obtained as the main phase after heat treatment, and increasing the Li_3_PS_4_ content in the starting composition (*x*Li_3_PS_4_·Li_2_S·LiCl, *x* = 1.0–1.4) effectively suppressed the residual starting materials (Li_2_S and LiCl) resulting from the partial hydrolysis of Li_3_PS_4_ to Li_3_PO_4_ in aqueous media. The optimized sample (*x* = 1.2) exhibited a relatively high ionic conductivity of 1.2 × 10^–3^ S cm^–1^ at 25 °C and maintained stable cycling performance over 100 cycles in all-solid-state cells. Furthermore, this synthesis approach was successfully extended to other argyrodite-type compositions (Li_6_PS_5_Br and Li_6_PS_5_I), demonstrating its versatility. Importantly, this work demonstrates that argyrodite-type sulfide electrolytes, which have traditionally been considered difficult to synthesize using water, can be successfully prepared using water as the main solvent. This approach establishes a scalable and environmentally friendly method for high-performance sulfide solid electrolytes. Future work will focus on further reducing the impurities in the synthesized argyrodite-type materials and on extending this approach to other argyrodite-type electrolytes.

## Supplementary Material



## Notes

Comparison of structure and ionic conductivity of synthesized and commercial Li_6_PS_5_Cl; NMR and photograph of extracted THF phase; TG–MS of precursor powder; SEM images and EDX mappings of synthesized Li_6_PS_5_Cl; structure, ionic conductivity, and CHNS analysis of the synthesized Li_6_PS_5_Cl before and after heat treatment; LSV measurements of synthesized Li_6_PS_5_Cl; structure and ionic conductivity of Li_6_PS_5_X (X = Br, I); rate performance of all-solid-state cells using synthesized Li_6_PS_5_Cl; summary of ionic conductivities at 25 °C (PDF)

## References

[1] Sakuda A., Hayashi A., Tatsumisago M. (2013). Sulfide Solid
Electrolyte with Favorable
Mechanical Property for All-Solid-state Lithium Battery. Sci. Rep..

[2] Kato A., Nose M., Yamamoto M., Sakuda A., Hayashi A., Tatsumisago M. (2018). Mechanical
Properties of Sulfide Glasses in All-solid-state
Batteries. J. Ceram. Soc. Jpn..

[3] Kanno R., Hata T., Kawamoto Y., Irie M. (2000). Synthesis
of a New
Lithium Ionic Conductor, Thio-LISICON–Lithium Germanium Sulfide
System. Solid State Ionics.

[4] Hayashi A., Hama S., Morimoto H., Tatsumisago M., Minami T. (2001). Preparation of Li_2_S–P_2_S_5_ Amorphous Solid Electrolytes by Mechanical Milling. J. Am. Ceram. Soc..

[5] Miura A., Rosero-Navarro N. C., Sakuda A., Tadanaga K., Phuc N. H. H., Matsuda A., Machida N., Hayashi A., Tatsumisago M. (2019). Liquid-phase
Syntheses of Sulfide Electrolytes for All-solid-state Lithium Battery. Nat. Rev. Chem..

[6] Tanigaki H., Kimura T., Kurioka E., Motohashi K., Sakuda A., Tatsumisago M., Hayashi A. (2025). Liquid-phase Synthesis
of Li_4_GeS_4_ and Li_10_GeP_2_S_12_ Electrolytes using Water as the Main Solvent. Chem. Commun..

[7] Kimura T., Tanigaki H., Sakuda A., Tatsumisago M., Hayashi A. (2024). Aqueous Solution Synthesis of Lithium-ion
Conductive
Tin-based Sulphide Electrolytes. Green Chem..

[8] Hashii T., Tanigaki H., Sakashita H., Kowada H., Motohashi K., Sakuda A., Hayashi A. (2026). Synthesis of Li_10_GeP_2_S_12_ Electrolytes Using Aqueous Solution. J. Am. Chem. Soc..

[9] Deiseroth H.-J., Kong S.-T., Eckert H., Vannahme J., Reiner C., Zaiß T., Schlosser M. (2008). Li_6_PS_5_X: A
Class of Crystalline Li-Rich Solids with an Unusually High Li^+^ Mobility. Angew. Chem., Int. Ed..

[10] Rao R. P., Adams S. (2011). Studies of Lithium argyrodite Solid
Electrolytes for All-Solid-State
Batteries. Phys. Status Solidi A.

[11] Kraft M. A., Culver S. P., Calderon M., Böcher F., Krauskopf T., Senyshyn A., Dietrich C., Zevalkink A., Janek J., Zeier W. G. (2017). Influence of Lattice Polarizability
on the Ionic Conductivity in the Lithium Superionic Argyrodites Li_6_PS_5_X (X = Cl, Br, I). J.
Am. Chem. Soc..

[12] Adeli P., Bazak J. D., Park K.-H., Kochetkov I., Huq A., Goward G. R., Nazar L. F. (2019). Boosting
solid-state diffusivity
and conductivity in lithium superionic argyrodites by halide substitution. Angew. Chem., Int. Ed..

[13] Auvergniot J., Cassel A., Ledeuil J. B., Viallet V., Seznec V., Dedryvère R. (2017). Interface Stability of Argyrodite Li_6_PS_5_Cl toward LiCoO_2_, LiNi_1/3_Co_1/3_Mn_1/3_O_2_, and LiMn_2_O_4_ in
Bulk All-Solid-State Batteries. Chem. Mater..

[14] Boulineau S., Courty M., Tarascon J. M., Viallet V. (2012). Mechanochemical Synthesis
of Li-Argyrodite Li_6_PS_5_X (X = Cl, Br, I) as
Sulfur-Based Solid Electrolytes for All-Solid-State Batteries Application. Solid State Ionics.

[15] Rao R. P., Sharma N., Peterson V. K., Adams S. (2013). Formation and Conductivity
Studies of Lithium Argyrodite Solid Electrolytes Using In-Situ Neutron
Diffraction. Solid State Ionics.

[16] Yubuchi S., Teragawa S., Aso K., Tadanaga K., Hayashi A., Tatsumisago M. (2015). Preparation of High Lithium-ion Conducting
Li_6_PS_5_Cl Solid Electrolyte from Ethanol Solution
for All-solid-state
Lithium Batteries. J. Power Sources.

[17] Arnold W., Buchberger D. A., Li Y., Sunkara M., Druffel T., Wang H. (2020). Halide Doping Effect
on Solvent-Synthesized Lithium Argyrodites Li_6_PS_5_X (X= Cl, Br, I) Superionic Conductors. J.
Power Sources.

[18] Heo Y. J., Seo S., Hwang S., Choi S. H., Kim D. (2022). One-pot Aprotic Solvent-enabled
Synthesis of Superionic Li-argyrodite Solid Electrolyte. Int. J. Energy Res..

[19] Hwang S.-H., Seo S.-D., Kim D.-W. (2023). A Novel
Time-Saving Synthesis Approach
for Li-Argyrodite Superionic Conductor. Adv.
Sci..

[20] Subramanian Y., Rajagopal R., Ryu K.-S. (2021). High Ionic-Conducting
Li-Argyrodites
Synthesized Using a Simple and Economic Liquid-Phase Approach and
Their Application in All Solid-State-Lithium Batteries. Scr. Mater..

[21] Yubuchi S., Uematsu M., Hotehama C., Sakuda A., Hayashi A., Tatsumisago M. (2019). An Argyrodite
Sulfide-Based Superionic Conductor Synthesized
by a Liquid-Phase Technique with Tetrahydrofuran and Ethanol. J. Mater. Chem. A.

[22] Lee J. E., Park K.-H., Kim J. C., Wi T. U., Ha A. R., Song Y. B., Oh D. Y., Woo J., Kweon S. H., Yeom S. J., Cho W., Kim K., Lee H.-W., Kwak S. K., Jung Y. S. (2022). Universal Solution
Synthesis of Sulfide
Solid Electrolytes Using Alkahest for All-solid-state Batteries. Adv. Mater..

[23] Zhou L., Park K.-H., Sun X., Lalère F., Adermann T., Hartmann P., Nazar L. F. (2019). Solvent-Engineered
Design of Argyrodite Li_6_PS_5_X (X = Cl, Br, I)
Solid Electrolytes with High Ionic Conductivity. ACS Energy Lett..

[24] Tsukasaki H., Sano H., Igarashi K., Wakui A., Yaguchi T., Mori S. (2022). Deterioration process
of argyrodite solid electrolytes during exposure
to humidity-controlled air. J. Power Sources.

[25] Chen Y.-T., Marple M., Tan D., Ham S., Sayahpour B., Li W., Yang H., Lee J., Hah H., Wu E., Doux J., Jang J., Ridley P., Cronk A., Deysher G., Chen Z., Meng Y. (2022). Investigating
dry room
compatibility of sulfide solid-state electrolytes for scalable manufacturing. J. Mater. Chem. A.

[26] Naillou P., Boulineau A., De Vito E., Ramos R., Azaïs P. (2024). Evidencing
Phase Transformations of Li_6_PS_5_Cl Argyrodite
under Ambient Air and Dry Room Exposure. ACS
Appl. Mater. Interfaces.

[27] Muramatsu H., Hayashi A., Ohtomo T., Hama S., Tatsumisago M. (2011). Structural
Change of Li_2_S–P_2_S_5_ Sulfide
Solid Electrolytes in the Atmosphere. Solid
State Ionics.

[28] Momma K., Izumi F. (2011). VESTA 3 for Three-Dimensional
Visualization of Crystal, Volumetric
and Morphology Data. J. Appl. Crystallogr..

[29] Liu Z., Fu W., Payzant E. A., Yu X., Wu X., Dudney N. J., Kiggans J., Hong K., Rondinone A. J., Liang C. (2013). Anomalous High Ionic Conductivity
of Nanoporous β-Li_3_PS_4_. J. Am. Chem. Soc..

[30] Ohta N., Takada K., Sakaguchi I., Zhang L., Ma R., Fukuda K., Osada M., Sasaki T. (2007). LiNbO_3_-coated
LiCoO_2_ as Cathode Material for All Solid-state Lithium
Secondary Batteries. Electrochem. Commun..

[31] Sakuda A., Takeuchi T., Kobayashi H. (2016). Electrode
Morphology In All-solid-state
Lithium Secondary Batteries Consisting of LiNi_1/3_Co_1/3_Mn_1/3_O_2_ and Li_2_S-P_2_S_5_ Solid Electrolytes. Solid
State Ionics.

[32] Yubuchi S., Ito A., Masuzawa N., Sakuda A., Hayashi A., Tatsumisago M. (2020). Aqueous solution
synthesis of Na_3_SbS_4_-Na_2_WS_4_ superionic conductors. J. Mater. Chem. A.

[33] Pearson R. G. (1963). Hard and
Soft Acids and Bases. J. Am. Chem. Soc..

[34] Sahu G., Lin Z., Li J., Liu Z., Dudney N., Liang C. (2014). Air-stable,
High-conduction Solid Electrolytes of Arsenic-substituted Li_4_SnS_4_. Energy Environ. Sci..

[35] Hayashi A., Tatsumisago M., Minami T. (2000). Crystallization Process of Lithium
Oxysulfide Glasses. J. Non-Cryst. Solids.

[36] Ananthapadmanabhan K.
P., Goddard E. D. (1987). Aqueous
Biphase Formation in Polyethylene Oxide-Inorganic
Salt Systems. Langmuir.

[37] Souza R. L., Lima R. A., Coutinho J. A. P., Soares C. M. F., Lima A. ´.S. (2015). Novel aqueous two-phase systems based on tetrahydrofuran and potassium
phosphate buffer for purification of lipase. Process Biochem..

[38] Zhu G., Ren B., Zhou Q., Xiong J., Ma X., Zhao L., Jiang F., Yang X., Wang S. (2023). Outstanding Performance
of the Deep Eutectic Solvent-Based Aqueous Biphasic System Constructed
with Sodium Citrate for a Green Gold Separation. Langmuir.

[39] Zhao D.-Y., Ding B., Zhu C.-Y., Gong L., Duan F. (2024). Effects of
Inorganic Salts on the Phase Separation of Partially Miscible Solutes. Langmuir.

