# Supplementary material for: Liquid-Phase Synthesis of Lithium Argyrodite Sulfide Electrolytes Using Tetrahydrofuran and Water
Source: Langmuir. 2026 May 30;42(23):16081–8. doi: 10.1021/acs.langmuir.5c06765 (PMC13276912; doi:10.1021/acs.langmuir.5c06765)
Supplement: Supplementary file 1 [file la5c06765_si_001.pdf]

Supporting Information

Liquid-Phase Synthesis of Lithium Argyrodite Sulfide Electrolytes Using Tetrahydrofuran and Water

Takashi Hashii[1], Hayata Tanigaki[1], Tomohiro Furukawa[1], Hiroe Kowada[1], Kota Motohashi[1], Atsushi Sakuda[1] and Akitoshi Hayashi[1,2]*

[1]Department of Applied Chemistry, Graduate School of Engineering, Osaka Metropolitan University, 1-1 Gakuen-cho, Naka-ku, Sakai, Osaka 599-8531, Japan

[2]Institute for Materials Research, Tohoku University, 2-1-1 Katahira, Aoba-ku, Sendai, Miyagi 980-8577, Japan

[*]Corresponding author:

Atsushi Sakuda, E-mail address: saku@omu.ac.jp

Contents of Supporting Information

Figure S1. XRD patterns of the Li_6PS_5Cl samples obtained using $x$$Li_3PS_4$·$Li_2S$·LiCl ($x$ = 1.0–1.4) precursors and commercial Li_6PS_5Cl (Sigma-Aldrich). S4

Figure S2. ^{31}P NMR spectrum of the extracted THF phase. S4

Figure S3. ^{1}H NMR spectrum of the extracted THF phase: (a) Full spectrum, (b) Enlarged view of (a). S5

Figure S4. Photograph of the THF phase after extraction using a separatory funnel. S5

Figure S5. TG–MS profiles of the 1.2Li_3PS_4·Li_2S·LiCl precursor powder prior to heat treatment at 550 °C. S6

Figure S6. SEM image and EDX elemental mappings of the Li_6PS_5Cl samples obtained using $x$$Li_3PS_4$·$Li_2S$·LiCl ($x$ = 1.0–1.4) precursors. S6

Figure S7. (a) XRD patterns and (b) ^{31}P MAS NMR spectra of the 1.2Li_3PS_4·Li_2S·LiCl samples before and after heat treatment at 550 °C. S7

Figure S8. Temperature dependence of the ionic conductivities of the 1.2Li_3PS_4·Li_2S·LiCl samples before and after heat treatment at 550 °C. S7

Figure S9. Temperature dependence of the ionic conductivities of the Li_6PS_5Cl samples obtained using the 1.2Li_3PS_4·Li_2S·LiCl precursor and commercial Li_6PS_5Cl (Sigma-Aldrich). S8

Figure S10. LSV curves of the cell using solid electrolyte obtained with the 1.2Li3PS4·Li_2S·LiCl precursor: (a) oxidation and (b) reduction. S8

Figure S11. XRD patterns of Li_6PS_5X samples obtained using 1.2Li_3PS_4·Li_2S·LiX precursors: (a) X = Br, (b) X = I. S9

Figure S12. Temperature dependence of the ionic conductivities of the Li_6PS_5X (X = Cl, Br, I) samples obtained using 1.2Li_3PS_4·Li_2S·LiX precursors. S9

Figure S13. Rate performance of the all-solid-state cell using the solid electrolyte obtained with the 1.2Li_3PS_4·Li_2S·LiCl precursor. S10

Table S1. Ionic conductivity at 25 °C and the activation energy of the Li_6PS_5Cl samples obtained using $x$$Li_3PS_4$·$Li_2S$·LiCl ($x$ = 1.0–1.4) precursors. Pellet density, powder density, and relative density are also listed. S10

Table S2. Weight ratios of C, H, N, and S in the 1.2Li_3PS_4·Li_2S·LiCl sample before and after heat treatment at 550 °C, as determined by CHNS analysis. S11

Table S3. Ionic conductivity at 25 °C and the activation energy of the Li_6PS_5X (X = Cl, Br, I) samples obtained using 1.2Li_3PS_4·Li_2S·LiX precursors. Pellet density, powder density, and relative density are also listed. S11

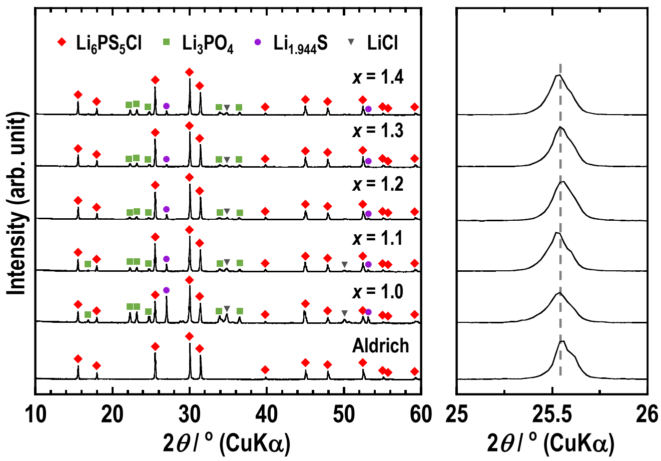

Figure S1. XRD patterns of the Li_6PS_5Cl samples obtained using $x$$Li_3PS_4$·$Li_2S$·LiCl ($x$ = 1.0–1.4) precursors and commercial Li_6PS_5Cl (Sigma-Aldrich).

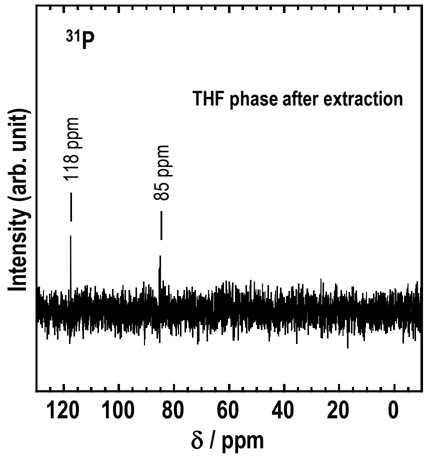

Figure S2. ^{31}P NMR spectrum of the extracted THF phase.

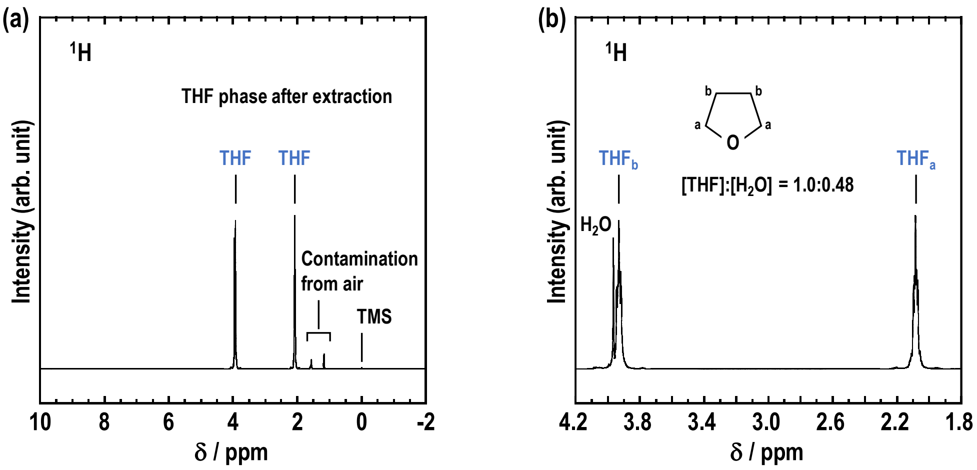

Figure S3. ^{1}H NMR spectrum of the extracted THF phase: (a) Full spectrum, (b) Enlarged view of (a).

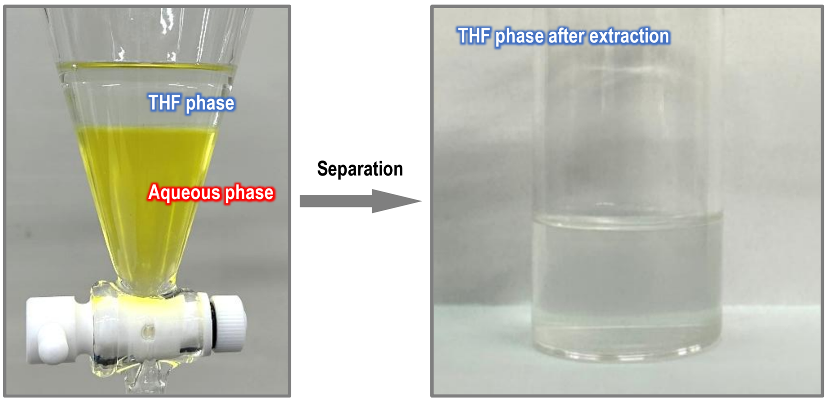

Figure S4. Photograph of the THF phase after extraction using a separatory funnel.

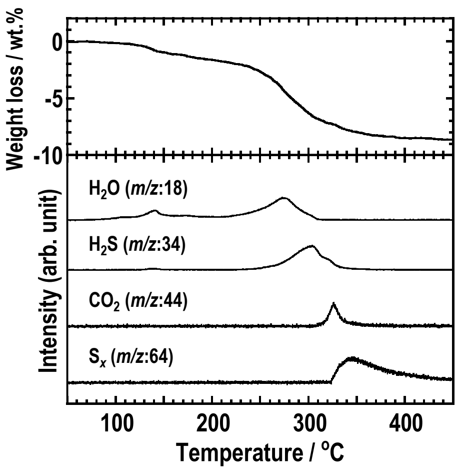

Figure S5. TG–MS profiles of the $1.2Li_3PS_4{\cdot}Li_2S{\cdot}LiCl$ precursor powder prior to heat treatment at 550 °C.

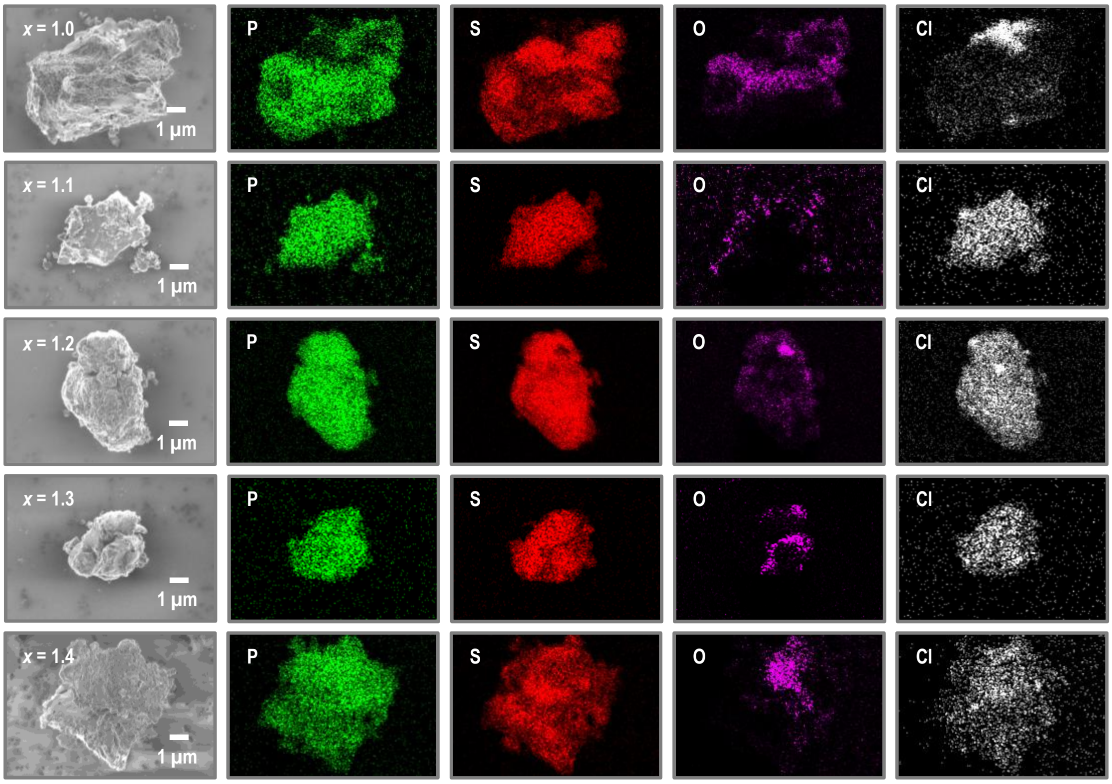

Figure S6. SEM image and EDX elemental mappings of the Li_6PS_5Cl samples obtained using $xLi_3PS_4{\cdot}Li_2S{\cdot}LiCl$ (x = 1.0–1.4) precursors.

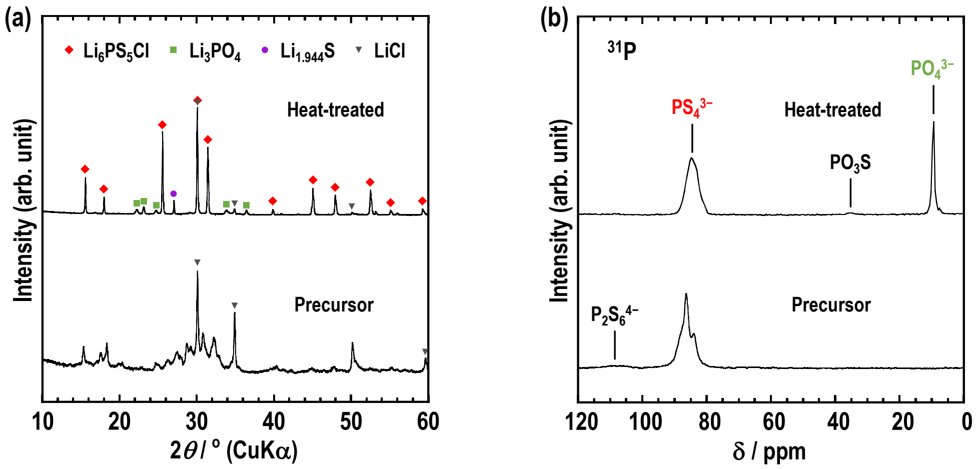

Figure S7. (a) XRD patterns and (b) ^{31}P MAS NMR spectra of the 1.2Li_3PS_4·Li_2S·LiCl samples before and after heat treatment at 550 °C.

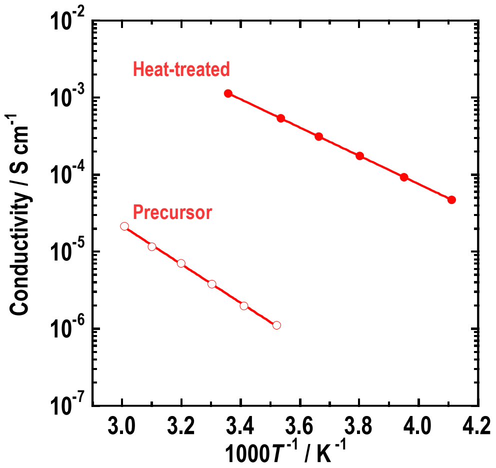

Figure S8. Temperature dependence of the ionic conductivities of the 1.2Li_3PS_4·Li_2S·LiCl samples before and after heat treatment at 550 °C.

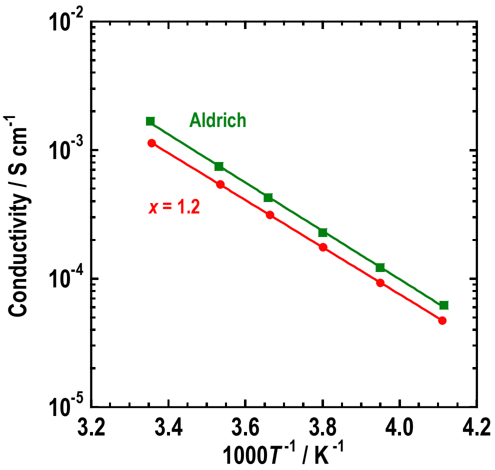

Figure S9. Temperature dependence of the ionic conductivities of the Li_6PS_5Cl samples obtained using the $1.2Li_3PS_4{\cdot}Li_2S{\cdot}LiCl$ precursor and commercial Li_6PS_5Cl (Sigma-Aldrich).

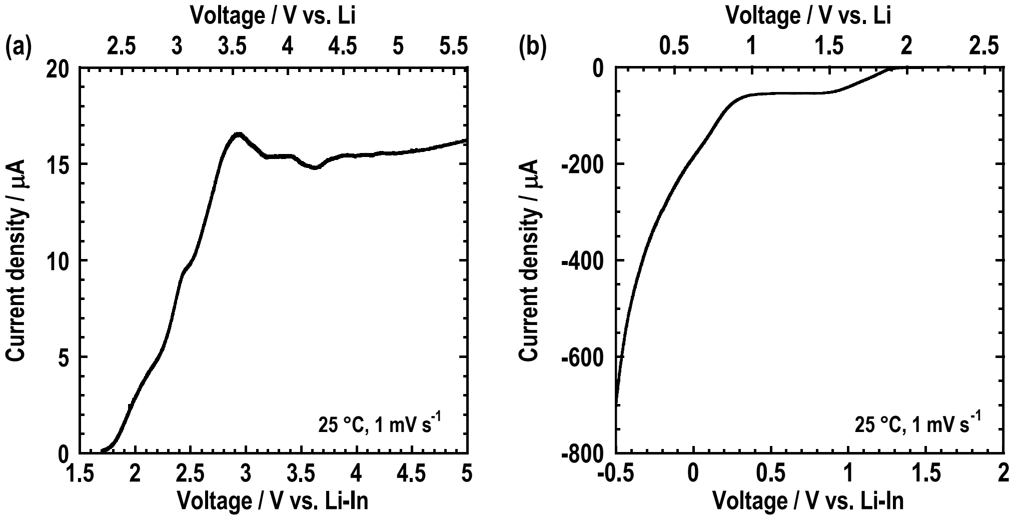

Figure S10. LSV curves of the cell using solid electrolyte obtained with the $1.2Li_3PS4{\cdot}Li_2S{\cdot}LiCl$ precursor: (a) oxidation and (b) reduction.

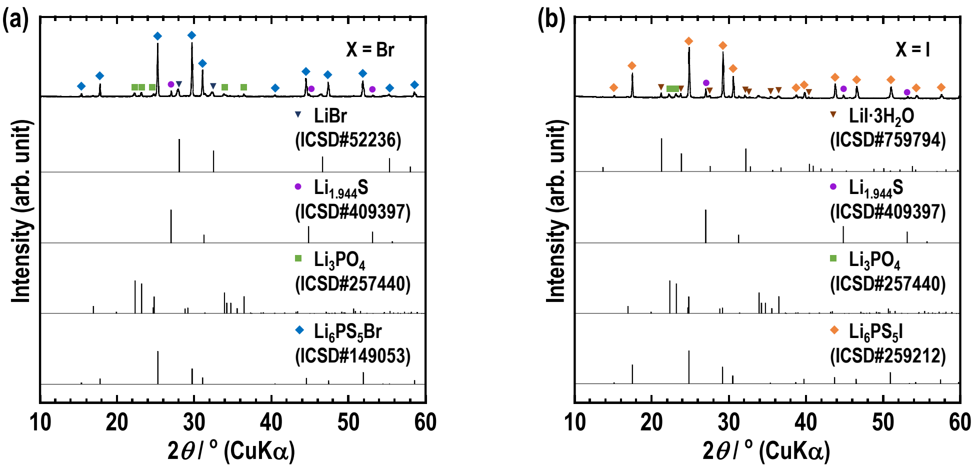

Figure S11. XRD patterns of Li_6PS_5X samples obtained using $1.2Li_3PS_4{\cdot}Li_2S{\cdot}LiX$ precursors: (a) X = Br, (b) X = I.

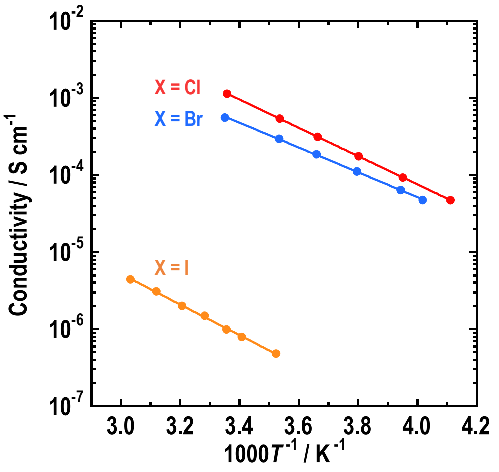

Figure S12. Temperature dependence of the ionic conductivities of the Li_6PS_5X (X = Cl, Br, I) samples obtained using $1.2Li_3PS_4{\cdot}Li_2S{\cdot}LiX$ precursors.

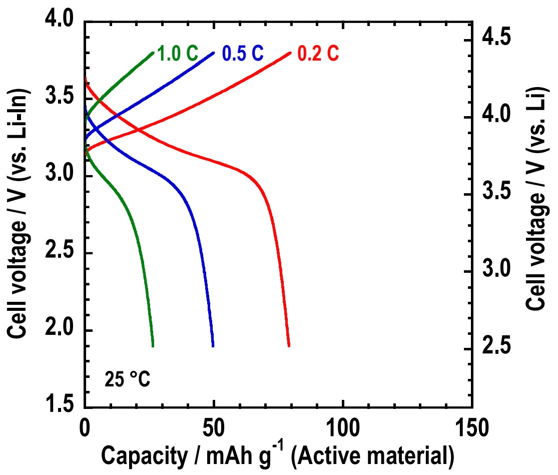

Figure S13. Rate performance of the all-solid-state cell using the solid electrolyte obtained with the $1.2Li_3PS_4 \cdot Li_2S \cdot LiCl$ precursor.

Table S1. Ionic conductivity at 25 °C and the activation energy of the Li_6PS_5Cl samples obtained using $xLi_3PS_4 \cdot Li_2S \cdot LiCl$ (x = 1.0–1.4) precursors. Pellet density, powder density, and relative density are also listed.

Composition	Ionic conductivity / S cm^{-1}	E_a / kJ mol^{-1}	Pellet density / g cm^{-3}	Powder density / g cm^{-3}	Relative density / %
$x = 1.4$	7.1×10^{-4}	35	1.58	1.98	80
$x = 1.3$	1.1×10^{-3}	35	1.59	1.96	81
$x = 1.2$	1.2×10^{-3}	35	1.50	1.93	78
$x = 1.1$	6.4×10^{-4}	37	1.62	1.96	83
$x = 1.0$	9.2×10^{-5}	37	1.58	2.02	78

Table S2. Weight ratios of C, H, N, and S in the $1.2Li_3PS_4 \cdot Li_2S \cdot LiCl$ sample before and after heat treatment at 550 °C, as determined by CHNS analysis.

Sample	N / wt%	C / wt%	H / wt%	S / wt%
Precursor (x = 1.2)	0.090	0.173	1.047	47.390
Heat-treated (x = 1.2)	0.027	0.050	0.647	44.233
Mortar-mixed starting materials	0.003	0.030	0.467	59.243
Theoretical value	0	0	0	61.100

Table S3. Ionic conductivity at 25 °C and the activation energy of the Li_6PS_5X (X = Cl, Br, I) samples obtained using $1.2Li_3PS_4 \cdot Li_2S \cdot LiX$ precursors. Pellet density, powder density, and relative density are also listed.

Composition	Ionic conductivity / S cm^{-1}	E_a / kJ mol^{-1}	Pellet density / g cm^{-3}	Powder density / g cm^{-3}	Relative density / %
X = Cl	1.2×10^{-3}	35	1.50	1.93	78
X = Br	5.6×10^{-4}	31	1.76	2.18	81
X = I	1.0×10^{-6}	38	1.72	2.27	76
